# Medical Opinion and Movement

**Published:** 1907-06-29

**Authors:** 


### Medical Opinion and Movement.

Mr. H. Rider Haggard, J.P., delivered an address to the students of St. Thomas's Hospital on Wednesday afternoon, June 26, on the occasion of their annual prize-giving. Subsequent speakers dilated on the advantage of hearing the views of one who is not a member of the medical profession, and especially one who brings to bear a breadth of mind only found in those who, like Mr. Haggard, have played many parts in strange countries. With this contention we have great sympathy, and anyone who listened to the well-known novelist's stirring words must acknowledge that the St. Thomas's authorities did well to prefer them to anything which could have fallen from a pillar of the profession, however eminent.

There is undoubtedly a growing conviction among the profession that a more thorough and complete organisation of the provident dispensaries of London offers one of the best solutions to the problem of the present abuse of the out-patient departments of the general hospitals. A conference of representatives of the provident dispensaries from all parts of the metropolis was recently held at the residence of Mr. Francis Buxton, Chairman of the Metropolitan Provident Medical Association, and a resolution was adopted to the effect that co-operation between all the provident dispensaries of London is desirable, in order that their work may be advanced and reciprocal action between them and hospitals promoted. A provisional committee was appointed to carry out the objects of the resolution, and to arrange for the appointment of a consultative council representing all the provident dispensaries of London. Such a central authority should prove useful in establishing them on a firm basis and giving them their proper position among London medical charities and institutions. Then we may hope that by some reciprocal arrangement with the hospitals the present abuse of hospital out-patients may at least in some measure be reformed.

The Professorial Chair of Anatomy at the Paris Faculty of Medicine has just been officially declared vacant, owing to the death of Professor Poirier a little time back. His successor will be appointed in about three weeks from now. But the Council of the Faculty, with a view to reform, have come to the conclusion that it is desirable for all professors of purely scientific aspects of medicine to devote their whole time to science, and, consequently, not to practise, medicine or surgery at all. A unanimous resolution was therefore passed by the Council that henceforward the functions of the professors of anatomy, physiology, histology, physics, chemistry, natural history, and materia medica are incompatible with those of a doctor or hospital surgeon. The professor will have to remain a professor, and leave the practice of medicine and surgery to practitioners. This change will be followed after a time by reform in other directions. The French medical profession is said to be solidly in favour of the new scheme, and the campaign which has been carried on in the Press against various shortcomings in the matter of method or practice has doubtless not been without some real use, in spite of the vigorous, if not exaggerated, terms in which these shortcomings have been condemned by some writers.

The Royal Medical Society, which has been formed by the union of fifteen of the London Medical Societies, held its inaugural meeting on June 14, when a code of by-laws was adopted and the officers and members of the Council were elected. It is perhaps to be regretted that the original scheme of a complete union of all the medical societies of London has not been carried through, owing to the preference of certain of the societies—notably the Medical Society of London—to remain independent; nevertheless, considering the many difficulties and the fruitless results of previous attempts in a similar direction, those who have worked for this object are certainly to be congratulated on the success they have obtained. One cannot but sympathise with the desire of members of a society with traditions and individuality to preserve its independence. At the same time the union of the many branches of medicine into one corporate body must offer many obvious advantages, both of an economic and social nature, and tend to a furtherance of general medical science. Every effort is being made to give to each section of the new society as much independence as is compatible with the administration of the whole, so that the individuality of the original societies may as far as possible be retained, and we have every hope therefore that they will not regret their absorption into the larger corporate body.

Some interesting observations have been recently made by Mr. A. Mouneyrat on the influence of motoring on the general nutrition of both healthy and anæmic subjects. The individuals experimented upon were taken for a motor run of 100 to 200 kilometres, at an average pace of 40 kilometres an hour, for eight to ten days. During this period it was found that the number of red corpuscles increased in large proportions, and also their hæmoglobin content. This occurred in the case of the anæmic subjects as well as those in normal health. Examination of the urine showed an excessive activity of the processes of metabolism, and there was a corresponding increase in the appetite. A further beneficial effect ascribed by the author to motoring is the influence on sleep. He finds that with normal subjects sleep is more prolonged, and that in the case of neurasthenics suffering from insomnia their power of sleep is completely restored. If these observations receive confirmation from other sources, the motor car should prove a useful therapeutic agent for these conditions, though we seem to be able to recall not a few anæmic and neurasthenic subjects among the motoring class. It may, however, be justly argued that motoring at random, accompanied probably by the irregularities of a life of pleasure, would have quite a different effect upon the health from motoring under certain definitely prescribed conditions.

